# Quantitative analysis of triangular fibrocartilage complex injury by 3.0T MR 3D VIBE and T2 mapping techniques

**DOI:** 10.1097/MD.0000000000031589

**Published:** 2022-12-23

**Authors:** Mei Yan, Shengbao Wen, Xueyan Wang

**Affiliations:** a Department of Medical Imaging Center, Qinghai University Affiliated Hospital, Xining, China.

**Keywords:** 3D VIBE, T2 mapping, triangular fibrocartilage

## Abstract

To evaluate triangular fibrocartilage complex (TFCC) injury based on quantitative analysis of fibrocartilage by 3.0T MR 3D VIBE and T2 mapping techniques. In this study, 25 patients with TFCC (15 patients with unilateral injury and 10 patients with bilateral injury) and 30 healthy subjects were enrolled. All 55 participants underwent bilateral wrist joint routine plain scan + enhanced scan, 3D VIBE and T2 mapping sequence scan. The 2 hands were divided into the dominant group and the non-dominant hand group. Pseudo-color images of T2 mapping were obtained through the post-processing workstation. Except for the meniscus homologue, there were statistical differences in the overall mean T2 value of all other regions between the injured group and the healthy group (*P* < .001). The T2 value of each region in the injury group was statistically different in the pairwise comparison (all *P* < .02). There was no statistical difference in the overall mean T2 value between the dominant hand group and the non-dominant hand group. 3.0T MR 3D VIBE and T2 mapping techniques are helpful for the evaluation of TFCC injury and the quantitative analysis of fibrocartilage. The parameters can reflect molecular changes of fibrocartilage injury, and T2 values are not affected by dominant hand, age and gender.

## 1. Introduction

The triangular fibrocartilage complex (TFCC) is composed of triangular fibrocartilage (TFC) and its surrounding ligaments.^[1]^ TFCC is the most important fibrocartilage-ligament complex structure on the ulnar side of the wrist, located on the ulnar side of the wrist joint, and it separates the ulnar wrist and the distal radioulnar joint, mainly stabilizes the joint and buffers the pressure, especially for the stability of the distal radioulnar joint. TFCC also plays an important role in bearing, buffering and transmitting the axial pressure of the wrist joint. When TFCC has a disordered structure or is damaged, ulnar wrist pain and wrist dysfunction will occur. At present, the TFCC injury classification system proposed by Palmer in 1989 is mostly used clinically.^[1]^ According to the pathological site, Palmer divided TFCC injury into traumatic (class I) and degenerative (class II); type IA: central perforation; type IB: ulnar tear (with or without ulnar styloid fracture); type IC: distal tear; Type ID: radial tear (with or without sigmoid notch fracture); type IIA: TFC abrasion; Type IIB: TFC abrasion + ulnar/lunate chondromalacia; type IIC: TFC perforation + ulnar/lunate chondromalacia; type IID: TFC perforation + ulnar/lunate chondromalacia + lunotriquetral ligament tear; type IIE: TFC perforation + ulnar/lunate chondromalacia + lunotriquetral ligament tear + ulnar wrist arthritis. Due to the complex structure of the carpal bones and ligaments of the wrist joint, short ligaments, thin fibrocartilage, and overlapping fibers, it is very difficult to measure the structure of the wrist joint.

With recent development of imaging techniques, magnetic resonance imaging (MRI) has become currently the best method for noninvasive determination of TFCC injury. T2 mapping technique can reflect molecular biological changes such as polysaccharide loss, collagen fiber disorder, and water reduction before macroscopic degeneration of fibrocartilage, and T2 values are determined to evaluate the structural integrity, tissue structure and water content of fibrocartilage. MRI is conducive to sensitive measurement of histological changes of fibrocartilage, and has high specificity, sensitivity and precision with great potential in clinical applications.^[2]^

In this study, MRI 3D VIBE and T2 mapping sequences were used to evaluate TFCC injury and other ligament and soft tissue injuries, and quantitative analysis of fibrocartilage, and actively identify joint pain caused by synovial hyperplasia and pannus formation caused by rheumatoid arthritis.^[3]^

## 2. Data and Methods

### 2.1. Clinical data

The data of 25 patients with TFCC injury (15 patients with unilateral injury and 10 patients with bilateral injury) treated at the Affiliated Hospital of Qinghai University due to wrist pain from January 2021 to April 2022 and 30 healthy subjects during the same period were collected. Among the patients with TFCC injury, 4 cases were male and 21 cases were female (aged 21–55 years, with an average age of 44.84 ± 56.26 years). Among the healthy subjects, 4 cases were male, and 26 cases were female (aged 20–60 years, with an average age of 37.60 ± 10.80 years).

Inclusion criteria: self-reported ulnar wrist pain, especially when exerting force, and clinically, and the tenderness near the ulnar styloid process of the wrist is obvious. Exclusion criteria: patients with benign and malignant tumors, congenital malformations and surgery at the wrist joint; those who cannot cooperate with the examination. All subjects signed informed consent form.

### 2.2. Instrument and methods

Siemens Prisma 3.0T magnetic resonance scanner with 15-channel high-resolution knee joint coil was used. In order to reduce the physiological differences of the bilateral wrist joints in different time periods, the subjects were instructed to be examined in the time period of 16:00 to 19:00. The subject was in the prone position, the upper forearms of the upper limbs were straightened over the head, the hands were combined, the back of the hand was fixed with a sandbag, and the forehead was cushioned with a sponge pad; the forearm was padded up to the shoulder level, and tied with an external fixation. The center of the coil was aligned with the wrist joint, the scanned wrist joint was brought close to the center of the magnetic field as far as possible, and the wrist was fixed so that it cannot move within the coil. All subjects underwent bilateral wrist joint routine plain scan + enhanced scan, VIBE sequence, and T2 mapping sequence scan, and conventional spin-echo (SE) sequence was used. VIBE sequence scan parameters: field of view: 220 mm, TR: 13.50 ms, TE: 6.00 ms, slice thickness: 0.5 mm, matrix: 238 × 256; scan parameters of coronal T2 mapping sequence: field of view: 160 mm, TR: 1510.0 ms, TE: 11.2 ms, slice thickness: 3.0 mm, matrix: 320 × 320.

### 2.3. Image processing

**Figure 1.**
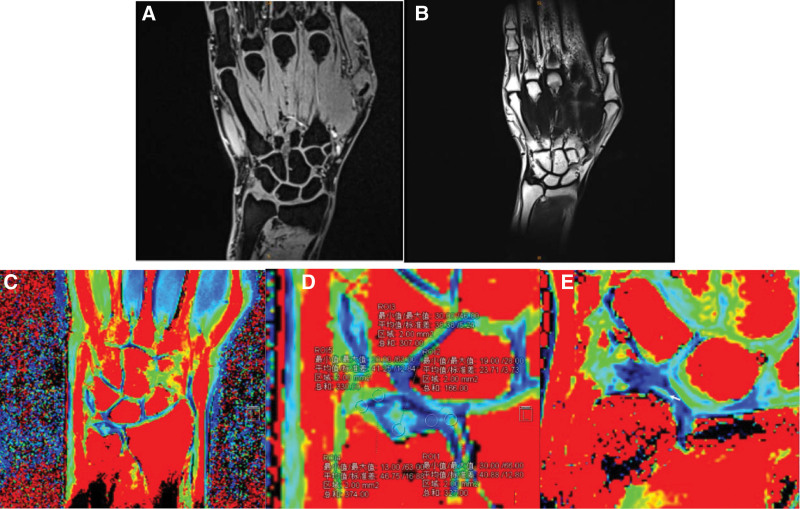
(A) Coronal 3D VIBE image of wrist joint; (B) original coronal T2 mapping image of wrist joint; (C) coronal T2 mapping pseudo-color image of wrist joint; (D) the regions of interest (ROIs) with a diameter of about 2 mm are delineated, ROI1 is radial cartilage, ROI2 is TFC, ROI3 is fibrovascular tissue, ROI4 is meniscus homologue, and ROI5 is ulnar attachment; (E) TFC injury, T2 signal is increased, and the color of TFC changes from dark blue to fluorescent green (as shown by the white arrow).

The original T2 mapping images were sent to the Prisma syngo.via post-processing workstation, and the pseudo-color images were generated through software analysis. Two physicians with experience in musculoskeletal imaging selected the maximum TFCC layer, and delineated the regions of interest (ROIs) with a diameter of about 2 mm on radial cartilage, TFC, fibrovascular tissue, TFC ulnar attachment, and meniscus homologue, respectively. The T2 values were automatically calculated by the software (Fig. 1). In order to reduce artifacts or misplaced ROIs, each ROI placement was repeated 3 times, and the average T2 value was used for further analysis.

### 2.4. Statistical analysis

SPSS 25.0 statistical software was used for processing and analysis, and the measurement data were expressed as x̅±s. The independent samples t-test was used to analyze the difference of T2 values between TFCC injury group and healthy group, and the difference of T2 values of TFCC injury patients between different genders. The paired t test was used to analyze the difference of T2 values of dominant hand and non-dominant hand. One-way analysis of variance was used to analyze the difference of T2 values in different types of TFCC injury and different age groups. The LSD-t test was used for the comparison between 2 groups.

## 3. Results

### 3.1. Comparison of T2 values between TFCC injury group and healthy group

A total of 25 patients with TFCC injury were enrolled, including 35 TFCC injury sites; 30 healthy subjects were enrolled, and 30 healthy TFCC sites were taken. The T2 values of radial cartilage, TFC, fibrovascular tissue, meniscus homologue and ulnar attachment in the TFCC injury group were (41.42 ± 2.81) ms, (39.03 ± 4.30) ms, (35.82 ± 3.80) ms, (46.14 ± 2.58) ms and (44.14 ± 2.93) ms, respectively, and those in the healthy group were (37.04 ± 0.85) ms, (27.78 ± 2.94) ms, (32.48 ± 1.35) ms, (46.34 ± 1.64) ms and (39.23 ± 0.96) ms, respectively. Except for meniscus homologue, there were statistical differences in the overall mean T2 values of all other regions between the injury group and the healthy group (*P* < .001) (Table 1). The T2 value of each region in the injury group was statistically different in the pairwise comparison (all *P* < .02).

**Table 1:** Comparison of T2 values of different regions of wrist cartilage between TFCC injury group and healthy group (ms, x̅±s).

Group	Radial cartilage	TFC	Fibrovascular tissue	Meniscus-like	Ulnar attachment
Injury group	41.42 ± 2.81	39.03 ± 4.30	35.82 ± 3.80	46.14 ± 2.58	44.14 ± 2.93
Healthy group	37.04 ± 0.85	27.78 ± 2.94	32.48 ± 1.35	46.34 ± 1.64	39.23 ± 0.96
t value	8.32	12.11	4.57	0.36	8.78
*P* value	<.001	<.001	<.001	.72	<.001

TCF = triangular fibrocartilage, TFCC = triangular fibrocartilage complex.

### 3.2. Comparison of T2 values between dominant hand group and non-dominant hand group

A total of 55 subjects were enrolled, and the dominant hand was determined by asking the subjects which hand they would use to write and eat (for the right wrist, n = 51; for the left wrist, n = 4). The average T2 value in dominant hand group was (30.66 ± 5.75) ms, and that in the non-dominant hand group was (31.40 ± 7.11) ms. There was no significant difference in the overall mean T2 value between dominant hand group and non-dominant hand group (the difference was 0.74, 95% CI was −1.30 to 1.78, t = 0.73, *P* = .47) (Table 2).

**Table 2:** Comparison of T2 values between dominant hand group and non-dominant hand group (ms, x̅±s).

Group	n	Mean ± standard deviation	Difference and 95%CI	*t* test	
				t value	*P* value
Dominant hand group	55	30.66 ± 5.75	0.74	0.73	.47
Non-dominant hand group	55	31.40 ± 7.11	(−1.30 to 1.78)		

### 3.3. Comparison of T2 values between different genders in TFCC injury

Among the 35 injury patients, there were 4 males and 31 females, and the T2 values were (40.58 ± 4.52) ms and (38.83 ± 4.30) ms, respectively; the overall mean T2 value between different genders was not statistically significant (t = −0.763, *P* = .451) (Table 3).

**Table 3:** Comparison of T2 values between different genders in TFCC injury (ms, x̅±s).

Gender	n	Mean ± standard deviation	Difference and 95%CI	*t* test	
				t value	*P* value
Female	31	38.83 ± 4.30	−1.76	−0.763	.451
Male	4	40.58 ± 4.52	(−6.43 to 2.92)		

TFCC = triangular fibrocartilage complex.

### 3.4. Comparison of T2 values of different types of TFCC injury

Among the 35 patients with TFCC injury, there were 9 cases of type IA, 4 cases of type IB, 12 cases of type IC, 2 cases of type ID, 1 case of type IIA, 1 case of type IIB, 1 case of type IIC, 1 case of type IID, and 4 cases of type IIE. Because the number of types IIA, IIB, IIC and IID is small, IIA, IIB, IIC, IID and IIE are classified as type II, with a total of 8 cases. The T2 values of type IA, IB, IC, ID and II were (39.27 ± 2.70) ms, (41.38 ± 6.34) ms, (37.98 ± 4.51) ms, (41.60 ± 3.05) ms and (38.52 ± 4.85) ms, respectively. There was no significant difference in the overall mean T2 values of the 5 sets of different types (F = 0.662, *P* = .663) (Table 4).

**Table 4:** Comparison of T2 values of different types of TFCC injury (ms, x̅±s).

Injury type	n	Mean ± standard deviation	*F* test	
			F value	*P* value
IA	9	39.27 ± 2.70	0.662	.663
IB	4	41.38 ± 6.34		
IC	12	37.98 ± 4.51		
ID	2	41.60 ± 3.05		
II	8	38.52 ± 4.85		

TFCC = triangular fibrocartilage complex.

### 3.5. Comparison and correlation analysis of T2 values at different age stages of TFCC injury

Among 35 patients with TFCC injury, there were 3 cases aged 20 to 29 years, 6 cases aged 30 to 39 years, 19 cases aged 40 to 49 years, and 7 cases aged 50 years and over. The T2 values were (32.43 ± 0.31) ms, (41.00 ± 2.99) ms, (39.63 ± 4.34) ms, and (38.54 ± 3.68) ms, respectively. There was a statistical difference in the overall mean T2 values at different ages (F = 3.611, *P* = .024) (Table 5). There was no significant difference in the correlation between the T2 value of TFCC injury and the age (*R* = 0.203, *P* = .243).

**Table 5:** Comparison of T2 values at different age stages of TFCC injury (ms, x̅±s).

Age (yrs)	n	Mean ± standard deviation	*F* test	
			F value	F value
20–29	3	32.43 ± 0.31	3.611	0.024
30–39	6	41.00 ± 2.99		
40–49	19	39.63 ± 4.34		
50 and over	7	38.54 ± 3.68		

TFCC = triangular fibrocartilage complex.

## 4. Discussion

Before the occurrence of fibrocartilage defects, the extracellular matrix will undergo significant changes, including increased water content, decreased proteoglycans and collagen fiber disintegration.^[4–6]^ The degeneration of TFCC is progressive and irreversible, which may eventually lead to perforation. Therefore, the method to visually observe TFCC injury and detect the early structural changes of biochemical components of TFC is not only helpful for early diagnosis, but also valuable for detecting the progression of the disease. In vivo noninvasive diagnosis of TFCC injury is usually difficult. With the steady improvement of MRI quality and the emergence of new sequence technique, the value of MRI in the diagnosis of TFCC injury is becoming obvious. T2 mapping is a new physiological fibrocartilage MRI technique, which can be used to evaluate the changes of articular cartilage matrix components and provide fibrocartilage metabolism and biochemical information.^[7]^ T2 mapping is a multi-echo SE technique. The T2 value of fibrocartilage is determined by the T2 relaxation time. T2 value of articular cartilage is related to the arrangement direction of cartilage collagen fibers, collagens, proteoglycans and water content, and can quantitatively reflect the changes of fibrocartilage components.^[8,9]^ T2 mapping technique has been applied in the studies on large joints such as sacroiliac joint, knee joint and shoulder joint,^[10,11]^ but there are relatively few related studies on small joints such as wrist joint.

In this study, 35 TFCC injuries and 30 healthy wrist joints of 55 subjects were scanned with T2 mapping. The T2 mapping pseudo-color images of the injury patients showed the color level change (from blue to fluorescent green) of fibrocartilage disk and the increase of T2 value. Felix et al and Mittal et al got the same conclusion and showed that the T2 value of fibrocartilage injury was higher than that of normal fibrocartilage.^[12,13]^ The increase of T2 value is related to the decrease of collagen fibers and proteoglycans and the increase of water content. Both human and animal experiments showed that the T2 value of fibrocartilage had a direct correlation with proteoglycans and water content.^[14]^ The T2 value of fibrocartilage is inversely proportional to the distribution of proteoglycans, but is proportional to the distribution of water.

In this study, the difference of T2 value of meniscus homologue between the injury group and the healthy group was not statistically significant. Studies have shown that meniscus homologue is the tissue in the ulnar joint capsule between the wrist disc and the ulnar styloid process, which is formed by the folding and thickening of the loose synovium, and its water content is high, similar to hyaline cartilage.^[15]^ At the same time, meniscus homologue is located at the ulnar edge of carpal cartilage, and is susceptible to the volume effect and the coil setting. Although the T2 value of injured fibrocartilage in the injury group will increase, there is no statistical significance between different types in the injury group, which may be related to the small sample size. Mikic et al showed that the degenerative changes of TFCC mostly started after the age of 30 and gradually become more frequent and severe with age.^[16]^ Our results are consistent with the histological results: the T2 value of the 20 to 29-year-old age group was (32.43 ± 0.31) ms, statistically significant compared to that of 30 to 39-year-old age group, 40 to 49-year-old age group, and 50-year-old and over age group. However, in this study there was no significant linear correlation between the T2 value of TFCC injury and the age (*R* = 0.203, *P* = .243), consistent with the results of Rauscher et al.^[17]^ The results of this study showed that the T2 value of TFCC injury between different genders was not statistically significant. Estradiol and progesterone exist in articular cartilage, and the estrogen content of women is higher than that of men, which can promote the proliferation of chondrocytes, resulting in the differences in the T2 value of shoulder cartilage between different genders.^[18]^

TFCC is a relatively small structure on the wrist joint, and its thickness is only a few millimeters. In most cases, TFC is about 14 to 16 mm in length and 9 to 11mm in width.^[19]^ The T2 value of fibrocartilage is susceptible to many factors, including the MRI system used, coil settings, spatial differences, volume effect, banded variation of cartilage, evaluated cartilage location, orientation of collagen fiber network relative to the magnetic field, magic angle effect that leads to the increase of signal intensity, component changes related to mechanical load, and different water contents.^[20–23]^ T2 mapping sequence is relatively sensitive to the magic angle effect, which easily leads to the extension of T2 relaxation time of fibrocartilage fibers in the direction of 55° with the magnetic field. In addition, Mars et al proposed that the difference of T2 value depended on the image type and calculation method of T2 mapping.^[24]^

Generally, the T2 values of knee meniscus and TFCC are considered to be similar, because they are both fibrocartilaginous structures, and their histological and MRI- related changes are expected to be similar. In this study, the average T2 value of the TFCC injury group and the healthy group was (39.03 ± 4.30) ms and (27.78 ± 2.94) ms, respectively. We do not necessarily expect a very similar age dependence, because all subjects are subjected to mechanical stress when walking and standing. In contrast, the mechanical stress of the hand-wrist depends more on personal behaviors, such as routine occupational and daily sports activities.

This study has several limitations. First, there are fewer male patients with TFCC injury, and the sample size needs to be further increased. Second, there was no significant difference in the T2 value of TFCC fibrocartilage between the dominant hand group and the non-dominant hand group, which may be due to small sample size. Third, manually drawn ROIs are used to evaluate 2-dimensional sequences, which are prone to human errors. It is expected that there will be more advanced technical methods in future, and high-resolution 3D T2 mapping can be used to automatically evaluate the whole fibrocartilage volume.

In summary, 3D Vibe technique can visually display the fibrocartilage morphology of the wrist joint, and the T2 mapping technique can quantitatively analyze the T2 value of the fibrocartilage, evaluate the early changes of the fibrocartilage components of the wrist joint, and provide meaningful help for the care of patients and the treatment of lesions.

## Author contributions

**Conceptualization:** Mei Yan, Shengbao Wen, Xueyan Wang.

**Data curation:** Mei Yan, Shengbao Wen, Xueyan Wang.

**Formal analysis:** Mei Yan, Shengbao Wen.

**Funding acquisition:** Mei Yan.

**Investigation:** Mei Yan, Shengbao Wen, Xueyan Wang.

**Methodology:** Mei Yan, Shengbao Wen, Xueyan Wang.

**Project administration:** Mei Yan, Shengbao Wen.

**Resources:** Shengbao Wen, Xueyan Wang.

**Software:** Mei Yan, Shengbao Wen, Xueyan Wang.

**Supervision:** Mei Yan, Shengbao Wen.

**Validation:** Shengbao Wen.

**Visualization:** Shengbao Wen.

**Writing – original draft:** Mei Yan, Shengbao Wen, Xueyan Wang.

**Writing – review & editing:** Shengbao Wen, Xueyan Wang.
